# Assessing microplastic pollution vulnerability in a protected coastal lagoon in the Mediterranean Coast of Egypt using GIS modeling

**DOI:** 10.1038/s41598-025-93329-2

**Published:** 2025-04-04

**Authors:** Muhammad A. El-Alfy, Hazem T. Abd El-Hamid, Amr E. Keshta, Abdelhamid A. Elnaggar, Dina H. Darwish, Afifi I. Basiony, Ahmad M. Alzeny, Marwa M. Abou-Hadied, Mohamed M. Toubar, Ahmed Shalby, Soha H. Shabaka

**Affiliations:** 1 National Institute of Oceanography and Fisheries, NIOF, Cairo, Egypt; 2 Faculty of Science, Tanta University, Tanta, Egypt; 3 Smithsonian Environmental Research Center, Edgewater, MD USA; 4 Faculty of Agriculture, Mansoura University, Mansoura, Egypt; 5 Faculty of Engineering, Tanta University, Tanta, Egypt

**Keywords:** Lake burullus, Microplastics, Land use, Non-point sources, GIS modelling, Environmental sciences, Ecological modelling, Wetlands ecology

## Abstract

Lake Burullus is a coastal wetland and protected area on the Mediterranean coast of Egypt. Despite the importance of this lake to the livelihood of millions of Egyptians, intensive anthropogenic activities and drainage water input have impacted its water quality. The current work aimed to evaluate the distribution and composition of microplastics (MPs) in Lake Burullus and analyze the impact of non-point pollution sources on their distribution and fate. The results showed that the average concentration of MPs in the open water was significantly lower (165.0 MPs/m^3^) than that near the drains (835.6 MPs/m^3^). The thermal analysis indicated the presence of eight thermoplastic polymers, originating from diffuse sources mainly via land-based activities. Moreover, a model was generated using ArcGIS 10.5 to assess the vulnerability of surface water to pollution from non-point sources. This model incorporated factors such as proximity to roads, waterways, land use, and urban areas, in addition to salinity and total suspended solids (TSS). Comprehensive maps were generated to visually illustrate the areas expected to be affected by MPs pollution. The results showed that waterways played an important role in in the transport of MPs to Lake Burullus. In addition, urban areas were identified as pollution hotspots, indicating the impact of land-use changes in urban areas. Salinity and TSS were also identified as important factors affecting the fate of MPs. Implementing strong measures to control land pollution in urban areas and managing wastewater inputs is highly recommended to effectively mitigate the impact of MPs on the ecosystem of Lake Burullus.

**Supplementary Information:**

The online version contains supplementary material available at 10.1038/s41598-025-93329-2.

## Introduction

Coastal wetlands are vital natural resources that provide ecosystem services such as water purification, habitats for biota, and blue carbon sequestration^1,2^. Coastal lagoons are biodiversity hotspots in the Mediterranean Sea, with a high numbers of endemic species^3^. However, urbanization, pollution, and industrial and agricultural activities significantly impact the services provided by coastal wetlands^4^. Lake Burullus, the second-largest lake in Egypt, holds immense importance and was designated as one of the world’s internationally significant wetlands under The Ramsar Convention in 1988. Despite its ecological value, Lake Burullus has been facing detrimental effects due to the escalating input of drainage water and the use of pesticides^5,6^.

Microplastics (MPs) have emerged as a concerning form of pollution, capturing the attention of numerous researchers due to their persistence in the environment^7–13^. These tiny particles, measuring less than 5 mm in size, can infiltrate the aquatic food chain leading to significant detrimental effects on the overall well-being of organisms^14,15^. The uptake of MPs can negatively impact the organisms at a molecular level, affecting gene expression and the generation of reactive oxygen species, at the cellular level, causing cell apoptosis and membrane stability, and at the population level, impairing development, reproduction, and feeding behavior^16^.

Several studies have reported the presence of MPs in coastal freshwater wetlands^14,17–21^. Wetlands tend to accumulate significant quantities of MPs in water and sediment, particularly, in the form of fibers and fragments^22^. For instance, in the Mangrove Forest of Beibu Gulf of China, water samples contained up to 5531 particles/m^3^, while sediment samples had 6360 particles/kg. Furthermore, wetland biota such as sea snails had MPs concentrations ranging from 7 to 53 particle/kg^23^.

Coastal lagoons are particularly susceptible to MPs pollution because of their shallow depth, limited water exchange, and elevated anthropogenic pressure^24^. The Nile Delta of Egypt is exposed to various sources of pollution such as wastewater treatment plants, agricultural runoff, and industrial effluents, making coastal lagoons highly susceptible to MPs pollution. Several studies have evaluated the negative impacts of MPs on the vitality of Nile tilapia (*Oreochromis niloticus*), a vital protein source for local communities in Egypt^25–28^. Additionally, high concentrations of MPs were detected in the sediment, water, and fish of the Mediterranean Deltaic coast of Egypt^29–32^. Moreover, Lake Burullus is among several major outlets along the Deltaic Mediterranean coast discharging about 38.8 million cubic meters of wastewater per year directly into the Mediterranean Sea coast^32^. Approximately 60% of the country’s fish production comes from Lake Burullus, particularly species with high economic value such as seabreams, seabass, and mullets, with the total annual catch exceeding 81,146 MT, according to fish statistics of the General Authority for Fish Resources Development of Egypt. Moreover, Lake Burullus was declared as a protectorate in 1998, and it is home to about 135 species of medicinal and fodder plants and a shelter for a large number of wild migratory birds. Unfortunately, coastal protected areas are particularly vulnerable to MPs pollution. Studies showed that organisms within coastal protected areas face numerous risks when they ingest plastic particles and transfer them through the food web. This process adversely impacts their resilience and biodiversity due to their exposure to a variety of toxins^33^. Consequently, there is an urgent need for understanding the main drivers and fate of plastic pollution in the lake to put adequate management plans that lessen the impact of discharged water into the eastern Mediterranean basin.

This study aimed to investigate the distribution and characteristics of MPs in Lake Burullus and develop a predictive model to evaluate the impact of land use and environmental factors on the distribution and fate of MPs.

## Materials and methods

### Study site

**Fig. 1:**
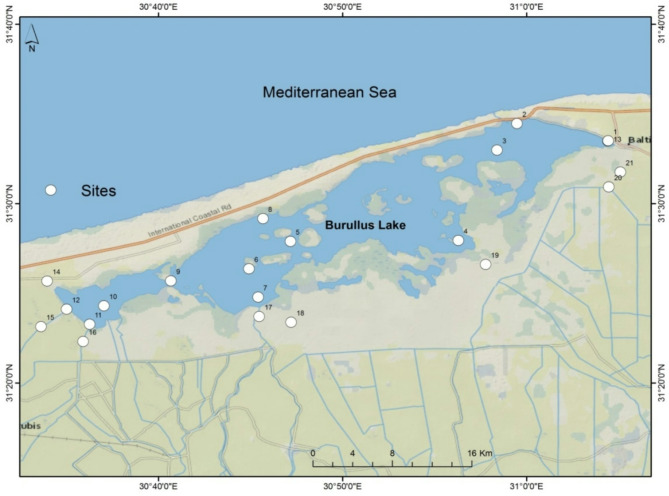
Location map of Lake Burullus and drains network with sampling stations overlaid. (Map was made using ArcGIS 10.5).

Lake Burullus covers an area of 455 km^2^ on the central part of the Deltaic Coast of Egypt. This lake is located in an arid region characterized by elevated air temperatures in summer, ranging from 30 to 35 °C, and warm winter averages between 10 and 20 °C, with an annual mean surface water temperature of 22.3 ± 5.2 °C (Fig. 1). Lake Burullus is very shallow with depths ranging from 0.6 m to 1.5 m, reaching a maximum depth of 5 m at the connection with the Mediterranean Sea (Elboughaz). Various sources of agricultural and industrial wastewater flow into Lake Burullus, particularly, in the southern regions^6,34^. Moreover, the water exchange between the lake and the Mediterranean Sea, via Elboughaz, has created a pollution and salinity gradient, leading to increased salinity and reduced pollution levels in the northern areas as opposed to lower salinity levels and heightened pollution concentrations in the southern area of the lake^6,35,36^.

According to Rifaat et al.^37^, the hydrodynamics of Lake Burullus are influenced by wind speed and direction, fluctuations in drain-water discharges, and daily water exchange through the Elboughaz. In the current study, sampling was conducted in November to explicitly assess the impact of anthropogenic factors on MPs pollution load, rather than the fate related to natural processes.

Twenty-one stations were sampled, covering the whole lake (Fig. 1; Table 1). The air temperature ranged from 21 to 24 °C, with no precipitation occurred, and the wind speed ranged from 0 to7 knots/hour (Table S1).

**Table 1:** Coordinates of the sampling stations in lake burullus.

No	Location	Name	Latitude	Longitude
1	Lake Burullus (open water)	Sothern the coastal road	31.55831	31.07369
2		Elrobaa village	31.57433	30.99122
3		Wish 7	31.54972	30.97319
4		Awal Elghab	31.46558	30.93819
5		West Elboughaz	31.46481	30.78611
6		Elhouis	31.43922	30.74858
7		Brinbal	31.41303	30.75678
8		Elhoks	31.48583	30.76117
9		Eltal	31.42806	30.67783
10		Elshaklouba	31.40486	30.61719
11		Elfasal Elgharby	31.38764	30.60425
12		Elboughaz	31.40175	30.58344
13	Drains	East Elburulls	31.07369	31.55830
14		West Elburullus	30.56589	31.42789
15		Brinbal	30.56076	31.38485
16		Elhoks	30.59812	31.37162
17		Elshaklouba	30.75811	31.39477
18		Damru	30.78640	31.38911
19		Drain 7	30.96282	31.44383
20		Tirrah	30.06568	31.51534
21		Elkashaa	31.08451	31.52892

### Microplastics sampling and analysis

100 L (0.1 m^3^) of surface water was collected using a Niskin bottle at 21 sampling stations. The sampled water was filtered through a plankton net, which had a mesh size of 55 μm, mouth aperture of 40 × 40 cm, and length of 1 m. The filtered water was concentrated in the cod end, and then emptied into closed glass vials^29,32,38^. At the laboratory, 10% KOH (v/v) was added to each sample to digest organic matter. The samples were then kept in an oven incubator at 50 °C for 3 days. The digested samples were then filtered on cellulose nitrate membrane filter (0.45 μm), using a vacuum pump. The filters were then examined under a stereomicroscope, and MPs were counted and classified based on their sizes, shapes, and colors.

The polymeric composition of the extracted MPs was determined using Differential Scanning Calorimetry (DSC) (Perkin-Elmer model-4000) according to the validated protocol developed by Shabaka et al.^32,39^ and El-Sayed et al.^31^. Extracted MPs from adjacent stations were pooled into a single sample, resulting in a total of 9 samples. The samples were placed in aluminum crucibles and subjected to thermal analysis under inert nitrogen with a flow rate of 20 mL/min and a heating rate of 10 ºC/min. The temperature range was set from 30 to 350 ºC and heat flow and temperature calibrations were performed using Indium and Zn standard melts.

### Quality control and contamination prevention

Measures were implemented for contamination control to ensure the accuracy and reliability of the MPs extraction and subsequent analysis. During the sampling, the Niskin bottle and the plankton net were washed thoroughly with double-distilled water prior to sampling at each station. All analyses were carried out within a laminar flow hood to minimize the risk of airborne contamination. To further minimize contamination, researchers wore cotton lab coats and utilized stainless steel or glass materials throughout the process. These materials underwent a thorough cleaning procedure involving rinsing twice with double-distilled water, followed by wiping with acetone. Observations were made in blank samples, which were run in sets of four for each extraction. To assess potential contamination, the filters were carefully examined under a stereomicroscope. During this assessment, a few instances of airborne contamination were observed, specifically white cotton fibers, which were examined under DSC and did not show a melting peak, suggesting natural cellulose. It is important to note that the types of MPs recovered from the actual samples were not observed in any of the blank samples.

### Statistical analysis

The stations were categorized into two groups representing surface water of the lake (Stations 1 to 12) and drains (Stations 13 to 21). To test if the data significantly deviated from a normal distribution (*p* < 0.05), Shapiro-Wilk and Levene’s tests were implemented. When the data showed non-normal distribution, it was transformed using Log_10_ (x + 1) to stabilize the variance, and then subjected to One-Way ANOVA to evaluate whether there were significant variations in MP concentrations between drains and surface water of the lake. The results were represented by the F-statistic and the associated p-value, where the null hypothesis was rejected when the significance level was less than 0.05. The statistical analysis was conducted using Statistica Software (Version: 12- StatSoft Inc.).

### Salinity and total suspended solids (TSS) measuring

The maps of salinity and total suspended solids (TSS) were generated using Landsat OLI-8 imagery (https://earthexplorer.usgs.gov/), based on Ansari and Akhoondzadeh^40^ and Ouma et al.^41^. The analysis utilized three bands: Band 1, which represents Coastal Aerosols (wavelength 0.43–0.45 μm); Band 2, Blue (wavelength 0.45–0.51 μm); and Band 3, Green (wavelength 0.53–0.59 μm). These bands, with a spatial resolution of 30 m, were processed using the raster calculator tool in ArcGIS 10.5 (Eqs. 1 and 2).

1 $$\:\text{T}\text{S}\text{S}\:\left(\text{m}\text{g}/\text{l}\right)=2454.1\text{*}\:{\text{B}}_{3}/{\text{B}}_{2}\:-1584.4$$

2 $$\:\text{W}\text{a}\text{t}\text{e}\text{r}\:\text{s}\text{a}\text{l}\text{i}\text{n}\text{i}\text{t}\text{y}\left({\upmu\:}\text{s}/\text{c}\text{m}\right)=570.80+26535.17\text{*}{\text{B}}_{1}+62141.71\text{*}{\text{B}}_{2}+34952.89\text{*}{\text{B}}_{3}$$

where, B_1_: Coastal Aerosols Band, B2: Blue Band, B3: Green Band.

### Water susceptibility to pollution model using GIS

Water susceptibility to pollution based on a non-point sources model was built according to UMD^42^ and Al-Adamat^43^. The latter authors estimated pollution based on nonpoint sources by incorporating of gradient slope, distance to water, land cover, and soil properties. In order to investigate the degree of water susceptibility to pollution by MPs in Lake Burullus, the factors were modified to include: distance to roads, distance to waterways, distance to land uses (residential areas, fish farms, farmlands, and industrial sites), distances to surrounding places (urban areas), in addition to two environmental factors, namely, salinity and TSS. The workflow adopted in the current work is presented in Fig S1.

In ArcToolbox of ArcGIS 10.5, the Euclidean distance analysis was conducted using safe distances from the factors of interest^44^. However, Euclidean distance calculations assume straight-line distances between points, potentially leading to some inaccuracies. The output of the analysis was raster maps with radial distances from the factors of interest, which were then reclassified and ranked from 1to 5, indicating a very low to very high effect (Table S2).

### Weighting of each factor using the analytic hierarchy process

The weight of each factor was determined by the Analytic Hierarchy Process (AHP), which is widely used as a decision-making method^45^. AHP constructs a hierarchy of decision items by evaluating the comparisons between each pair of factors, depending on the judgments of experts and these are then represented in a matrix^46^ (Table S3). Through these paired comparisons, weighting scores are generated to indicate the degree of significance or importance that each item holds in relation to one another (Table S4). In this process, the interpretation of eigenvalues is employed to calculate the consistency ratio, which is a measure of the consistency of the decision-making process^47^. Eigenvalues serve as indicators of the importance of each factor in relation to the overall decision. By quantifying the eigenvalues, the AHP method allows for the assignment of appropriate weights to each factor, reflecting their relative influence on the decision-making process (Fig. S2). Moreover, the consistency ratio provides an assessment of the degree of consistency in the decision-making process to measure the extent to which the assigned weights align with the underlying decision criteria. A lower consistency ratio indicates a higher level of consistency in the decision-making process, which enhances the reliability and validity of the calculated weights.

## Results and discussion

### Concentrations and distribution of microplastics

**Fig. 2:**
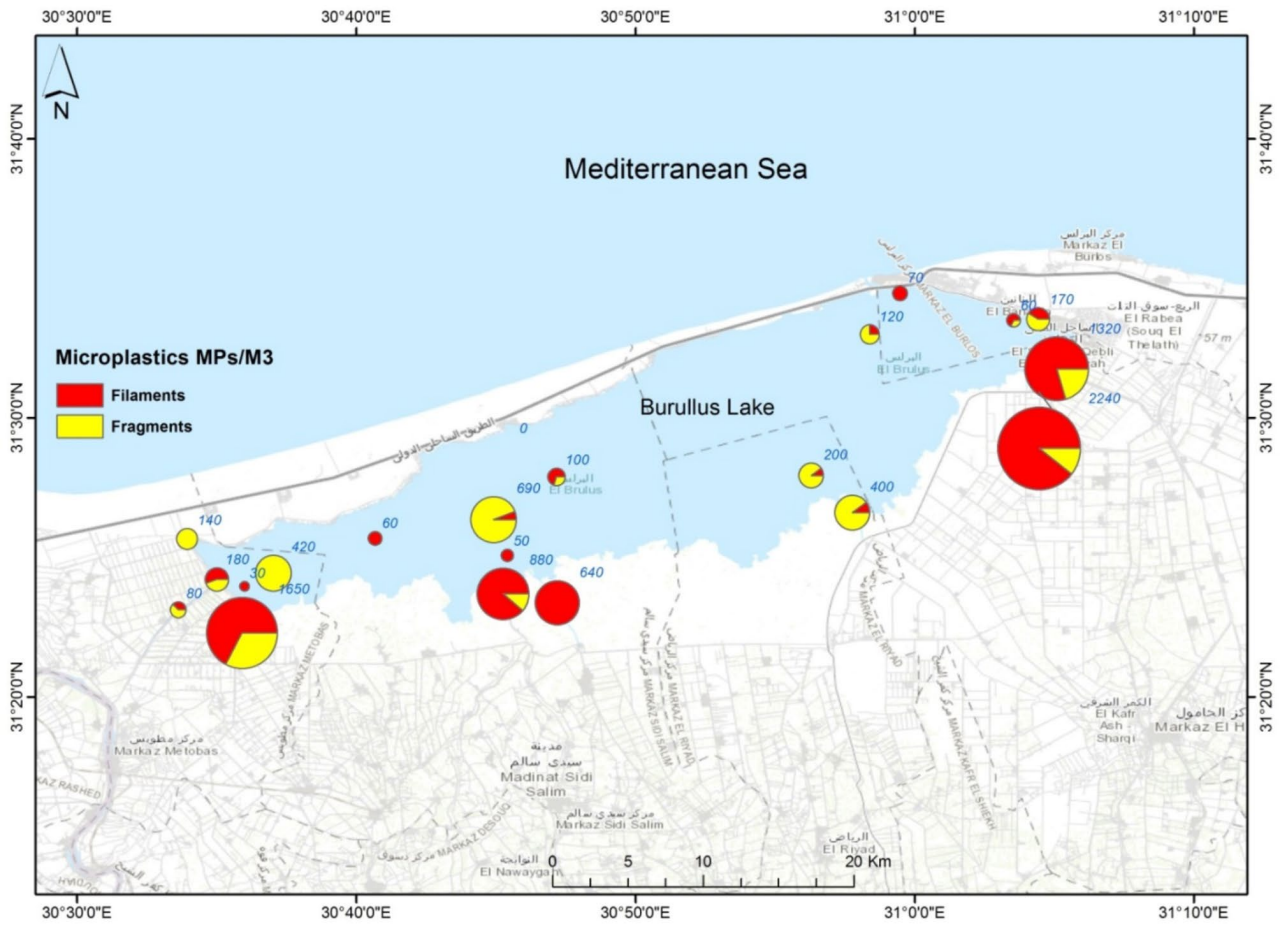
Concentrations and shapes of microplastics in Lake Burullus. (The distribution map was made using ArcGIS 10.5).

A total of 950 MPs were extracted from the surface water of Lake Burullus, with a mean concentration of 452.4 ± 606.2 MPs/m^3^. The open water of the lake exhibited a significantly lower mean concentration (165.0 ± 199.6 MPs/m^3^) compared to surface water near the drains (835.6 ± 758.1 MPs/m^3^) (F = 134.6, *P* = 0.00). MPs were detected in all stations, except for station 8. More than 60% of the stations had concentrations of ≤ 200 MPs/m^3^ (Fig. 2). The highest values were recorded at drains 20, 16, and 21 (2240, 1650, and 1320 MPs/m^3^, respectively), followed by drain 17 (880 MPs/m^3^) (Fig. 2). For the lake surface water, stations 6 and 10 had higher concentrations than the other stations (690 and 420 MPs/m^3^) (Fig. 2).

MPs’ concentrations were moderate in comparison to concentrations reported in other coastal lagoons worldwide^24^ (Table S5). MP concentrations ranged between 0 items/ m^3^ in a protected marine area in Sri Lanka^48^, < 1 items/m^3^ in coastal lagoons of Brazil^49,50^, 44–51 items/m^3^ in Colombia^51^, 83–135 items/ m^3^ in French Polynesia^52^, 400–453 items/m^3^ in Tunisia^53,54^, up to 208,000 items/m^3^ in Nigeria^55^, and 24,000,000 items/m^3^ in Florida lagoon in USA^56^. It should be noted that the method of sampling and processing can significantly affect the results. For instance, as shown in Table S5 and reference therein, towing with a plankton net with large mesh size yields lower concentrations than bulk sampling with water samplers^52^. Moreover, the significant variability in MP concentrations reflects not only geographic and environmental factors but also the effectiveness of regional policies aimed at mitigating plastic pollution. Understanding these differences is crucial for developing targeted strategies to address MPs pollution in diverse aquatic ecosystems.

In the current study, a distinct pattern of MP distribution was observed around the lake. The concentrations of MPs showed a noticeable gradient, with stations located in the southern region, particularly those near the drains, exhibiting significantly higher levels of MPs compared to stations in the middle or northern sections of the lake. This pattern can be attributed to the increased discharge of industrial runoff and agricultural drainage in the southern and eastern parts of the lake, which serve as the main sources of heavy MPs pollution.

### Characteristics of microplastics

Secondary MPs, in the shape of filaments (fibers) and fragments, were reported in the current study (Fig. 2). As indicated in Table S5, both shapes are prevalent in coastal lagoons worldwide. Filaments were the dominant shape of MPs, particularly in the eastern and southern parts of the lake, representing 70–100% of the examined particles, where they showed different colors, including red, black, and white. Fragments mainly of blue, green, and glossy colors were dominant in the western part of the lake, ranging in occurrence from 60 to 100% (Fig. 2). The widespread existence of microfibers as a marine contaminant is alarming^57^, as they reach water bodies through various pathways, including wastewater discharge, deposition from the atmosphere, shipping, fishing activities, and domestic waste discharges containg textiles. It has been estimated that a significant quantity of fibers, ranging from 1,800 to 52,000 metric tons, enters the oceans annually from various sources^58^. In the current study, the presence of filaments is mainly associated with drainage and domestic water discharges. On the other, in the Deltaic coast of Egypt fragments were reported to be associated with the breakdown of larger items after improper waste disposal^29,32^.

**Fig. 3:**
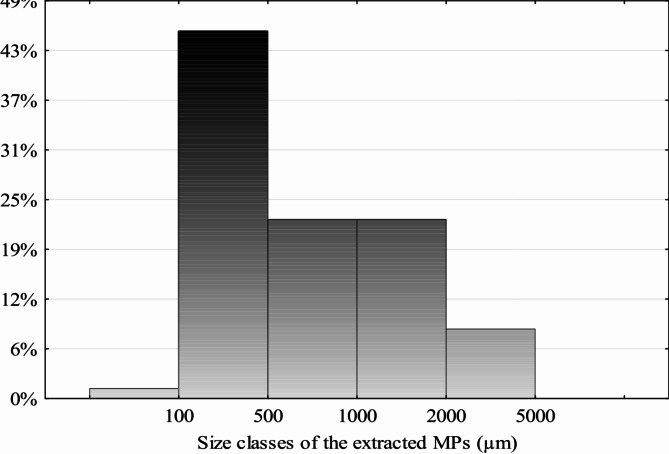
Size classes of microplastics extracted from surface water of Lake Burullus.

We conducted measurements on 800 randomly selected plastic particles. A wide range of sizes was recorded, spanning from 85 μm to 4,615 μm. The average size of these particles was determined to be 852.2 ± 969.7 μm, with a median size of 536 μm. It is noteworthy that approximately 45% of the MPs fell within the size range of < 100 μm to 500 μm (Fig. 3), making them easily ingested by a variety of marine organisms, including fish and invertebrates^31,59^. Exposure to MPs poses a significant threat to the well-being of Nile Tilapia (*Oreochromis niloticus*), particularly during their early juvenile stages^26^. Studies focusing on blood biomarkers indicate that MPs induce oxidative stress in the fish, leading to molecular damage^27^. Furthermore, research reveals that MPs trigger abnormal cell changes such as eryptosis and poikilocytosis, in young Nile Tilapia, potentially disrupting their physiological functions and overall health^28^. Moreover, small MPs of sizes around 200 μm can be adsorbed in the human body, as detected in human placenta and sputum^60,61^.

### Polymeric composition of microplastics

Eight synthetic polymers were identified in the samples, namely isotactic polypropylene (iPP), syndiotactic polypropylene (sPP), linear-low density polyethylene (LLDPE), low-density polyethylene (LDPE), polyamide/nylon (PA), polyethylene terephthalate (PET), polyethylene vinyl acetate (PEVA), and Polytetrafluoroethylene (PTFE) (Table 2). DSC thermograms are presented in Fig S3. LLDPE was commonly detected in most stations with percentage composition of more than 80%, followed by PP and PET (Table 2). The plastic polymers were more diverse in the eastern and middle sectors of the lake, whereas only three polymers, PEVA, PET, and LLDPE, were found in the western sector (Table 2). In the current study, the presence of eight plastic polymers with different applications indicates diffuse sources of pollution, particularly in the eastern part of the lake. Land-based activities seem to play a major role in MP pollution in Lake Burullus including agriculture runoff, industrial waste, urban runoff, and constructions. MPs pollution originates mainly from land-based sources compared to sea-based sources^62–64^. PE is the dominant polymer in water bodies, with a prevalence of 54.5%, followed by PP and PET (16.5% and 9.7%, respectively), originating mainly from land-based activities^65^. In the present work, LLDPE was frequently detected in all stations, which is commonly used in plastic bags, packaging, and agricultural films, indicating improper waste disposal and agricultural and urban runoffs^29^. Another PE isomer, LDPE, is used in various applications, including single-use plastic bags, food packaging, and disposable containers^32^. Its presence in the lake water may arise from littering and industrial discharges. Moreover, PP is widely used in packaging, textiles, consumer products, and fishing ropes^66,67^. iPP is known for its exceptional durability, which has led to its extensive use in marine-related applications, such as ropes and fishing nets^68^. sPP, another isomer of PP, is a thermoplastic elastomer that has superior properties and is commonly used as an insulating material in electric cables^69^; indicating industrial waste. PET is commonly used in beverage bottles, food containers, and polyester fibers^29^. Pollution with PET originates from the improper disposal and littering, and from domestic sewage discharges^70^. PTFE, commonly known as Teflon, is used in non-stick coatings, electrical insulation, and plumbing. Its presence in the lake suggests pollution from industrial sources^59,71–74^. Finally, PEVA is a polymer used in various consumer products, and most likely comes from boat paints^75,76^.

**Table 2:** Plastic polymers detected by DSC in the sampling stations and their corresponding melting points (T°C). The percentage composition of each polymer is presented between brackets.

Stations	Sector	LDPE	sPP	PA	PEVA	iPP	PTFE	LLDPE	PET
1,13	Eastern						320 (3%)	119 (96%)	284 (1%)
3,2			143 (1%)			165 (2%)		121 (96%)	
20,21								117 (100%)	
4,19		109 (9%)						121 (91%)	
5	Middle			186 (2%)		161 (12%)		117 (86%)	
17,7,6				182 (2%)		154 (1%)		124 (97%)	
18				222 (1%)	101 (8%)			124 (87%)	276(1%)
16,11,10,9	Western				86 (100%)				
12,15,14								117 (97%)	290 (3%)

### Modeling surface water susceptibility index to pollution by microplastics (SWSI-MPs)

The SWSI-MPs was estimated based on AHP analysis and the dependent factors of distance to waterways (WW), distance to land uses (LU), distances to surrounding places (PL), distance to roads (RO), salinity (Sal), and total suspended solids (TSS) (Eq. 3). The equation was applied using raster calculator of ArcToolbox in ArcGIS 10.5 (Al-Adamat, 2018)^77^.

3 $$\:\text{S}\text{W}\text{S}\text{I}-\text{M}\text{P}\text{s}=\text{W}\text{W}\text{*}58.8+\text{R}\text{O}\text{*}6.7+\text{P}\text{L}\text{*}8.1+\text{L}\text{U}\text{*}10.3+\text{S}\text{a}\text{l}\text{*}8.1+\text{T}\text{S}\text{S}\text{*}8.1$$

**Fig. 4:**
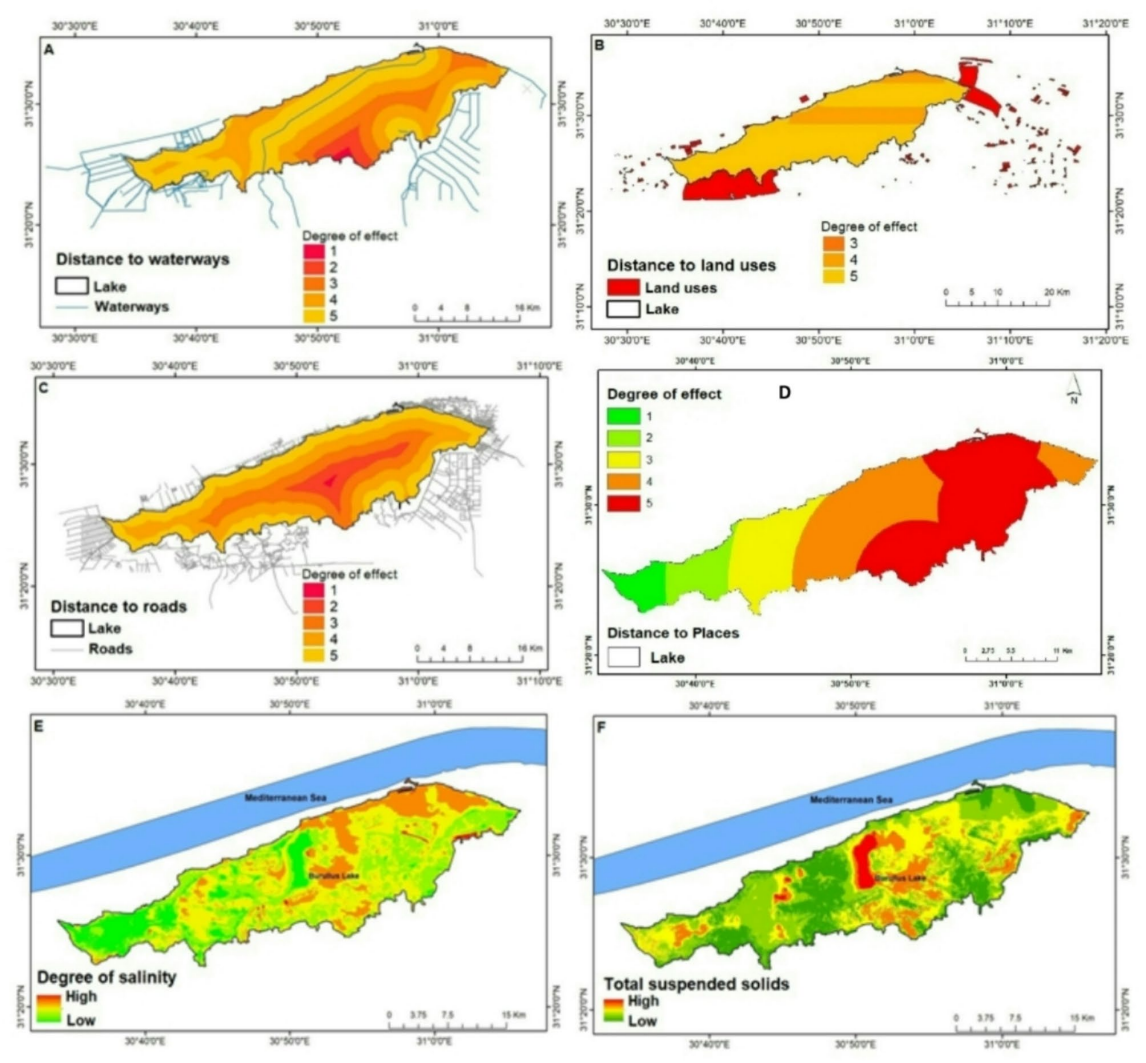
Euclidean distance for factors affecting MPs pollution in Lake Burullus. (**A**) Distance to water ways, (**B**) Distance to land uses, (**C**) Distance to roads, (**D**) Distances to surrounded places and environmental factors, (**E**) distribution of salinity, (**F**) distribution of total suspended solids. (Maps were made using ArcGIS 10.5).

**Fig. 5:**
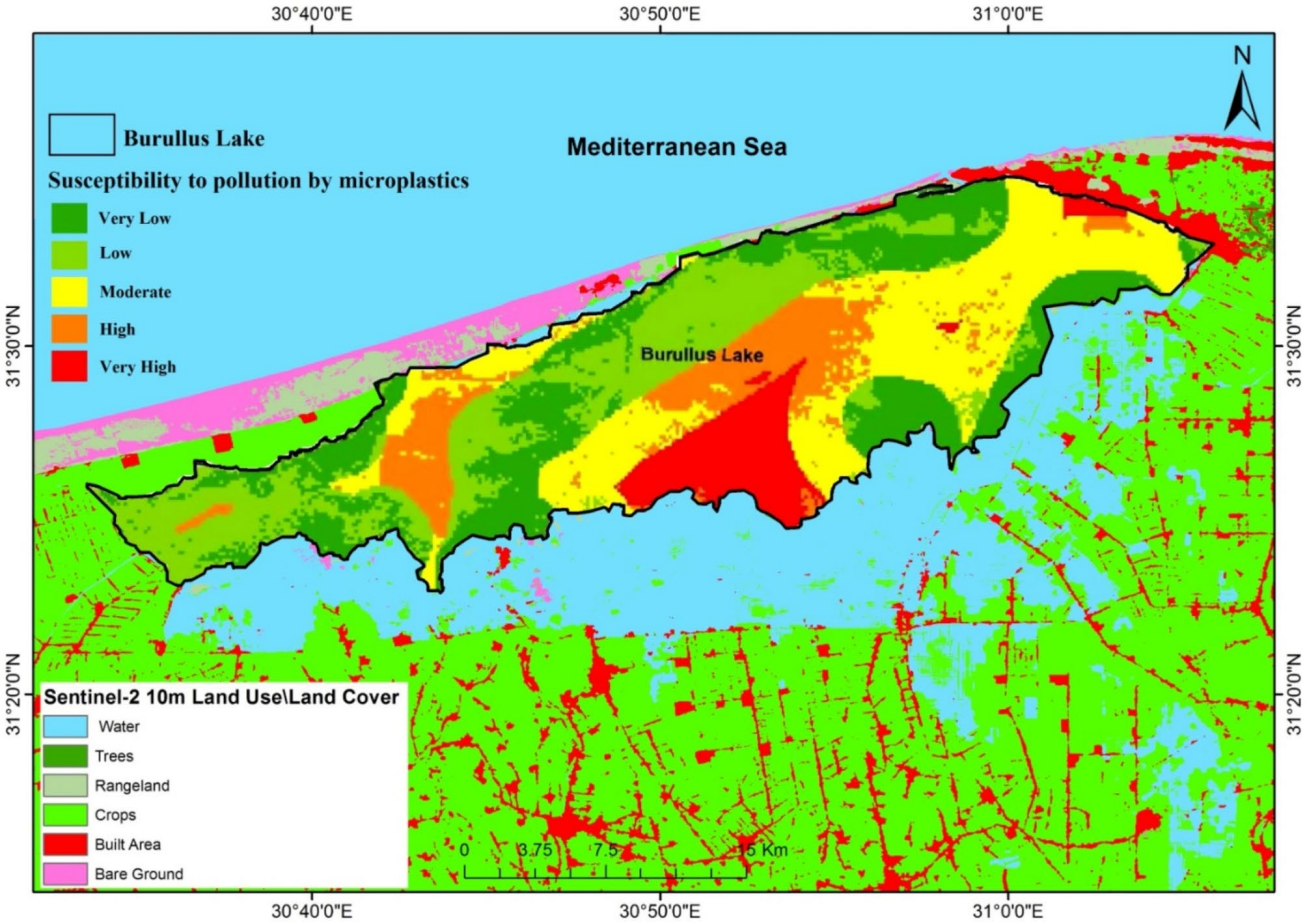
Final map of surface water susceptibility to microplastics pollution in Lake Burullus based on GIS model. (Source: this map was made using ArcGIS 10.5).

Figure 4A–F illustrates the spatial distribution of the factors included in the model. The final results of the model clearly indicate that the central-southern region of the lake, particularly near Elshaklouba and Drain 7, is highly susceptible to MPs (Fig. 5). The southern parts of the lake contain extensive waterways, including drains, canals, and small ditches. The study findings highlight that canals and drains are the main pathways for MPs. Lake Burullus receives approximately 3.9 billion m^3^ of agricultural runoff, drainage water, and discharges from fish farms annually^6^. In addition, the southern parts of the lake experience a notably high concentration of MPs, where urbanized areas such as Baltim, Alborg, and the southern-eastern parts show the highest concentrations. Urban areas are known to be hotspots for MPs pollution, where runoff largely contributes to aquatic pollution^78^. Previous studies have shown that urban regions exhibit higher concentrations of MPs, while public open spaces exhibit a negative correlation with MPs pollution^14^.

Residential areas, fish farms, farmlands, and industrial sites are the main land uses in Lake Burullus. Changes in land use / land cover (LULC) influence the transportation of MPs to water bodies^79^. Land use is recognized as a significant factor contributing to MPs pollution^80^. The intensity of land use has a strong correlation with MPs pollution, although this correlation decreases as the buffer radius (representing a greater distance) increases^81^. Therefore, the impact of land use in this study is assessed as ranged from moderate to high. Fragmented MPs are predominantly found in close proximity to farmland activities^82^.

Chen et al.^83^ have identified sewage disposal, irrigation, and proximity to roads as key factors associated with high levels of MP pollution. Additionally, several studies have shown that roads are significant sources of MPs^84^. Plastic litter from road activities is directly or indirectly reaching waterways. In this study, the road layer consists of different types of branched, primary, and secondary roads. The most influential road class was found to be tracking, followed by path. Rødland^85^ has suggested that the three main sources of MPs from roads and traffic are observed from the road surface, tire wear, and road marking.

Apart from the sources of MPs, environmental factors also play a significant role in the fate of MPs. An intriguing phenomenon, as discovered by Ye and Pei^81^, is that MPs sink when they bind to minerals and organic matter, as well as when aquatic organisms ingest them. Moreover, the water salinity was observed to affect the abundance of MPs, which is linked to the density and movement of water layers^86^. Salinity can affect the fate of MPs in Lake Burullus, where a decreasing salinity gradient from the connection to the Mediterranean Sea impacts the buoyancy and vertical distribution of MPs. In areas with higher salinity, where water density is greater, MPs tend to sink when they bind to minerals and organic matter. In Lake Burullus, the presence of TSS can significantly influence the concentration and fate of MPs. TSS acts as carriers for MPs, with higher TSS concentrations notably found in the central and eastern sectors of Lake Burullus, especially near drainage areas. The adsorption of MPs onto TSS particles leads to an increase in MP concentration within the water column, affecting their transport and overall fate within the lake. Moreover, TSS plays a crucial role in the sedimentation of MPs, facilitating their settling to the lake bed; however, the shallowness of the lake facilitates the re-suspension of MPs. Finally, land use changes and water pollution were found to affect ecosystem services of the Nile Delta Coastal Lakes^87^.

## Conclusion

The current study provided information on the distribution and characteristics of MPs in Lake Burullus and developed a predictive model to evaluate the impact of land use and environmental factors on their distribution and fate. The findings emphasize the importance of addressing non-point sources of pollution, particularly in urban areas and areas experiencing land use changes. Efforts to mitigate MP pollution should consider the role of industrial and agricultural drainage water as conduits for MPs. The generated maps indicating areas susceptible to MP pollution provide valuable insights for targeted management and conservation strategies in Lake Burullus. It is crucial to implement measures to reduce the input of MPs into the lake, safeguarding its ecosystem and maintaining its status as an internationally significant wetland. Additionally, it is important to define management approaches to mitigate plastic pollution effectively in the Mediterranean coastal lagoons to control the transmission of plastic litter into the Mediterranean basin.

Plastic pollution is primarily linked to waste generation in the Deltaic region of Egypt, which is closely linked to several critical issues as follows:

1. The narrow urban space surrounding the lake contributes to high waste generation in densely populated neighborhoods. Urban expansion further complicates waste management by increasing the distances between waste disposal sites and generation points, straining transportation resources.
2. There is an urgent need for clear policies defining the roles of both the private and informal sectors, alongside a well-structured institutional framework capable of planning and implementing effective waste management programs. Weak legislation and inadequate recycling facilities exacerbate the situation, limiting compliance with environmental standards.
3. The lack of intermediate receiving stations and suitable landfill sites compliant with environmental regulations hinders the effective transfer and disposal of waste. Moreover, existing recycling plants are often inefficient and fail to meet health and safety standards.
4. The lack of cohesive industries tailored to local waste specifications and market conditions further complicates waste management efforts.

To effectively address plastic pollution and enhance waste management practices, it is essential to develop comprehensive strategic goals and work programs for an integrated waste management system involving all levels of government and the private sector. Key targets should focus on improving collection efficiency, increasing recycling rates to at least 70% of total waste generated, and generating energy from waste to support energy-intensive industries like cement and iron. Additionally, measures must be implemented to prevent garbage accumulation and incineration in urban and rural areas, while addressing illegal dumping by closing existing sites. Establishing a sustainable administrative, financial, and technical framework for solid waste management is crucial, alongside applying the “polluter pays” principle to ensure financial independence and reduce the burden on the Egyptian government budget, making those responsible for pollution accountable for their contributions to mitigation and management costs.

## Electronic supplementary material

Below is the link to the electronic supplementary material.

Supplementary Material 1

## Data Availability

The datasets used and/or analysed during the current study available from the corresponding author on reasonable request.
