# Author Correction: Assessing microplastic pollution vulnerability in a protected coastal lagoon in the Mediterranean Coast of Egypt using GIS modelling

**DOI:** 10.1038/s41598-025-00674-3

**Published:** 2025-05-13

**Authors:** Muhammad A. El-Alfy, Hazem T. Abd El-Hamid, Amr E. Keshta, Abdelhamid A. Elnaggar, Dina H. Darwish, Afifi I. Basiony, Ahmad M. Alzeny, Marwa M. Abou-Hadied, Mohamed M. Toubar, Ahmed Shalby, Soha H. Shabaka

**Affiliations:** 1 National Institute of Oceanography and Fisheries, NIOF, Cairo, Egypt; 2 Faculty of Science, Tanta University, Tanta, Egypt; 3 Smithsonian Environmental Research Center, Edgewater, MD USA; 4 Faculty of Agriculture, Mansoura University, Mansoura, Egypt; 5 Faculty of Engineering, Tanta University, Tanta, Egypt

Correction to: *Scientific Reports* 10.1038/s41598-025-93329-2, published online 04 April 2025

The original version of this Article contained an error in the spelling of the author Ahmed Shalby which was incorrectly given as Ahmed Shalaby.

The original Article has been corrected.

