# Supplementary material for: Assessing microplastic pollution vulnerability in a protected coastal lagoon in the Mediterranean Coast of Egypt using GIS modeling
Source: Sci Rep. 2025 Apr 4;15:11557. doi: 10.1038/s41598-025-93329-2 (PMC11971271; doi:10.1038/s41598-025-93329-2)
Supplement: Supplementary file 1 — Supplementary Material 1 [file 41598_2025_93329_MOESM1_ESM.docx]

# Supplementary Materials

# Submitted to [Scientific](https://link.springer.com/journal/11356) Reports

**Assessing Microplastic Pollution Vulnerability in a Protected Coastal Lagoon in the Mediterranean Coast of Egypt Using GIS Modeling**

Muhammad El-Alfy^1^ ([muhammad.elalfy@yahoo.com](mailto:muhammad.elalfy@yahoo.com) , [ma.elalfy@niof.sci.eg](mailto:ma.elalfy@niof.sci.eg) ),

Hazem Abd El-Hamid^1^([hazem_ecology@yahoo.com](mailto:hazem_ecology@yahoo.com)),

Amr Keshta^2^([akeshta@terpmail.umd.edu](mailto:akeshta@terpmail.umd.edu)),

Abdelhamid Elnaggar^3^([abdelhamid.elnaggar@gmail.com](mailto:abdelhamid.elnaggar@gmail.com)),

Dina Darwish^1^ ([marawan.dina@yahoo.com](mailto:marawan.dina@yahoo.com)),

Afifi Basiony^1^([abasiony38@yahoo.com](mailto:abasiony38@yahoo.com)),

Ahmad Alzeny^1^([ahmadalzeny@gmail.com](mailto:ahmadalzeny@gmail.com)),

Marwa Abou-Hadied^1^ ([rosalina_marwa@yahoo.co.uk](mailto:rosalina_marwa@yahoo.co.uk)),

Mohamed Toubar^1^([tobar26@yahoo.com](mailto:tobar26@yahoo.com)),

Ahmed Shalby^4^([ahmed.shalby@f-eng.tanta.edu.eg](mailto:ahmed.shalby@f-eng.tanta.edu.eg))

*Soha Hamdy Shabaka^1^ ([sh.shabaka@niof.sci.eg](mailto:sh.shabaka@niof.sci.eg))

^1^ National Institute of Oceanography and Fisheries, NIOF, Cairo, Egypt

^2^ Faculty of Science, Tanta University, Tanta, Egypt

^3^ Faculty of Agriculture, Mansoura University, Mansoura, Egypt

^4^ Faculty of Engineering, Tanta University, Tanta, Egypt

* Corresponding author: Muhammad Abdul-Hady El-Alfy ([muhammad.elalfy@yahoo.com](mailto:muhammad.elalfy@yahoo.com) , [ma.elalfy@niof.sci.eg](mailto:ma.elalfy@niof.sci.eg) )

**PL**

**WW**

**LU**

**RO**

**Sal**

**Euclidean Distance**

**Weighting using AHP**

**Raster Calculator**

**Output**

**TSS**

**Fig. S1**. Chart of weighting and scaling of factors incorporated in the model. Distance to water ways (WW), distance to land uses (LU), distances to surrounded places (PL), distance to roads (RO), salinity (Sal), and total suspended solids (TSS).


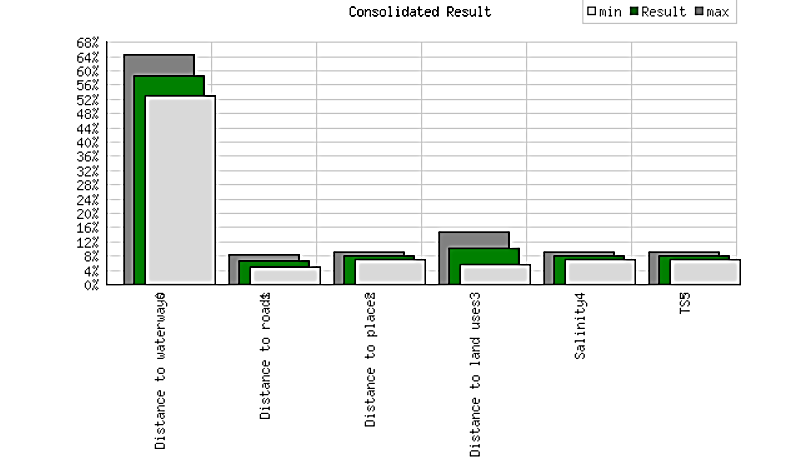


**Fig. S2** Priority percentages of the factors incorporated in the GIS model.

Fig. S3. DSC thermograms of the extracted microplastics

**4, 19**


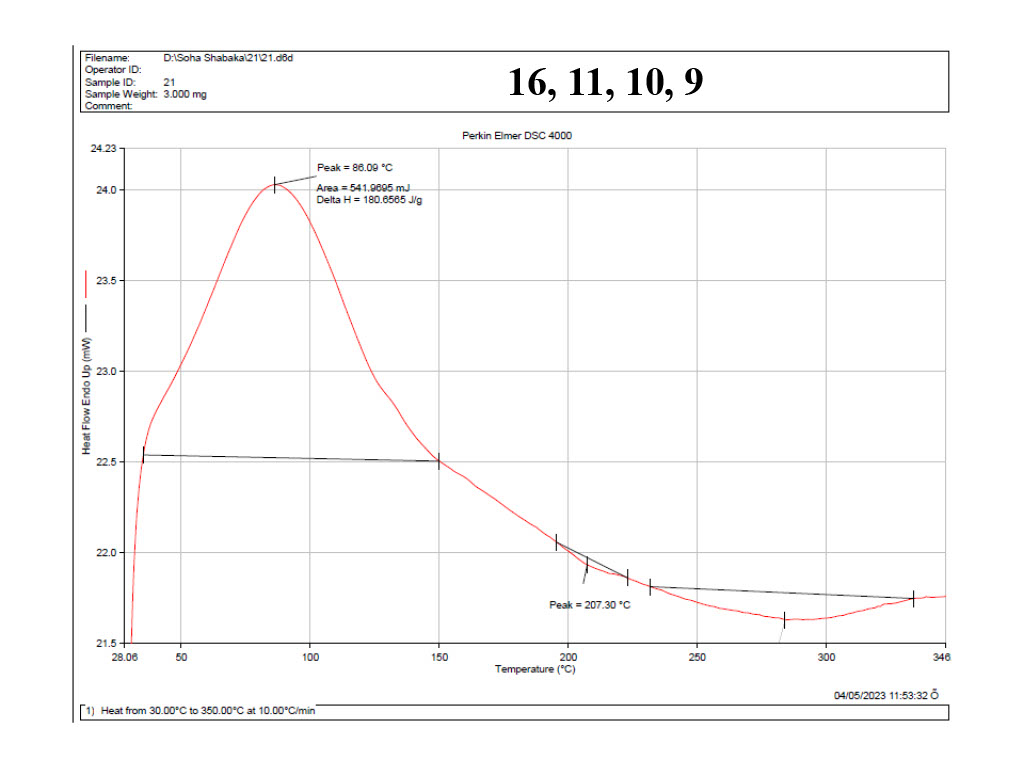

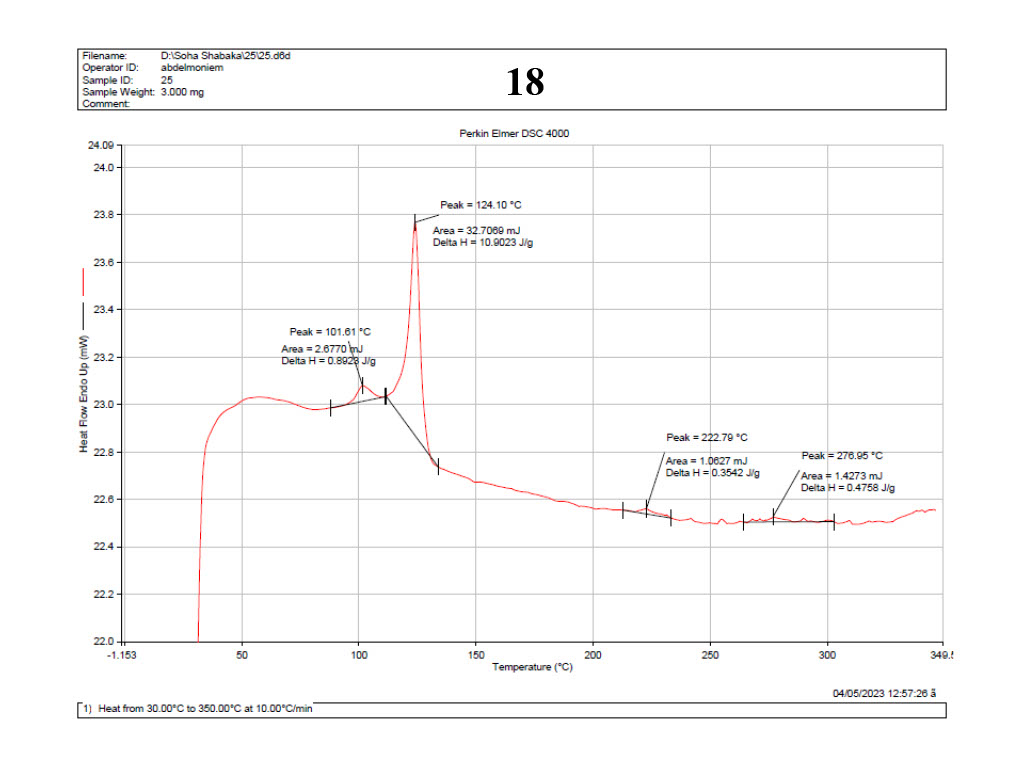

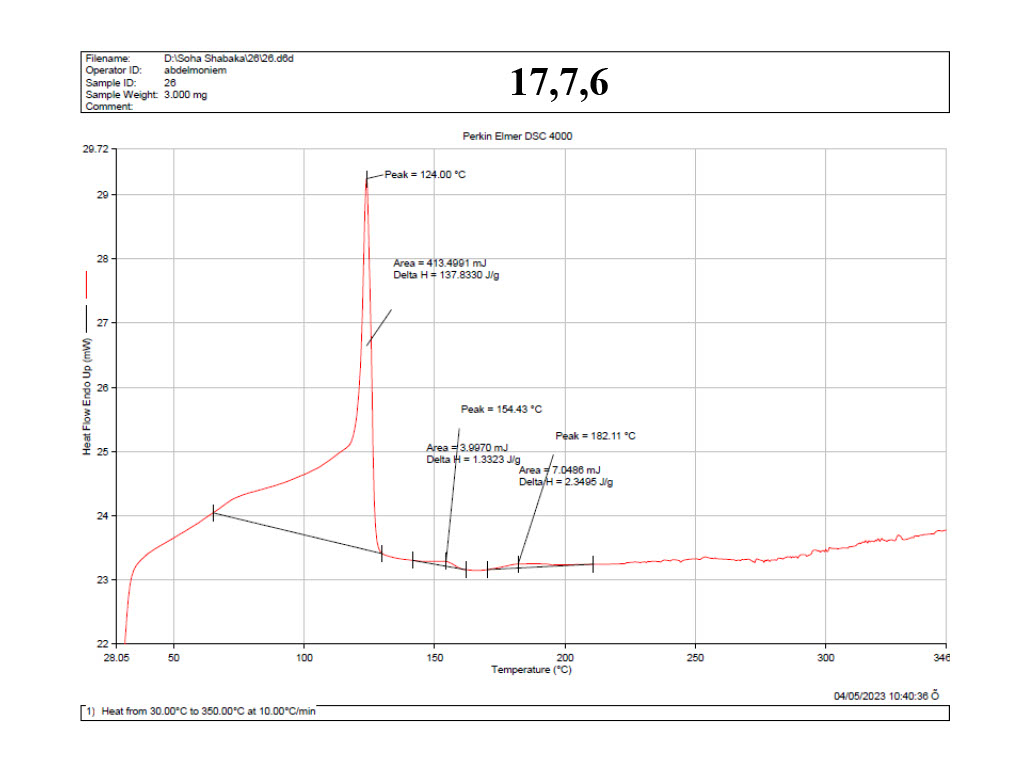

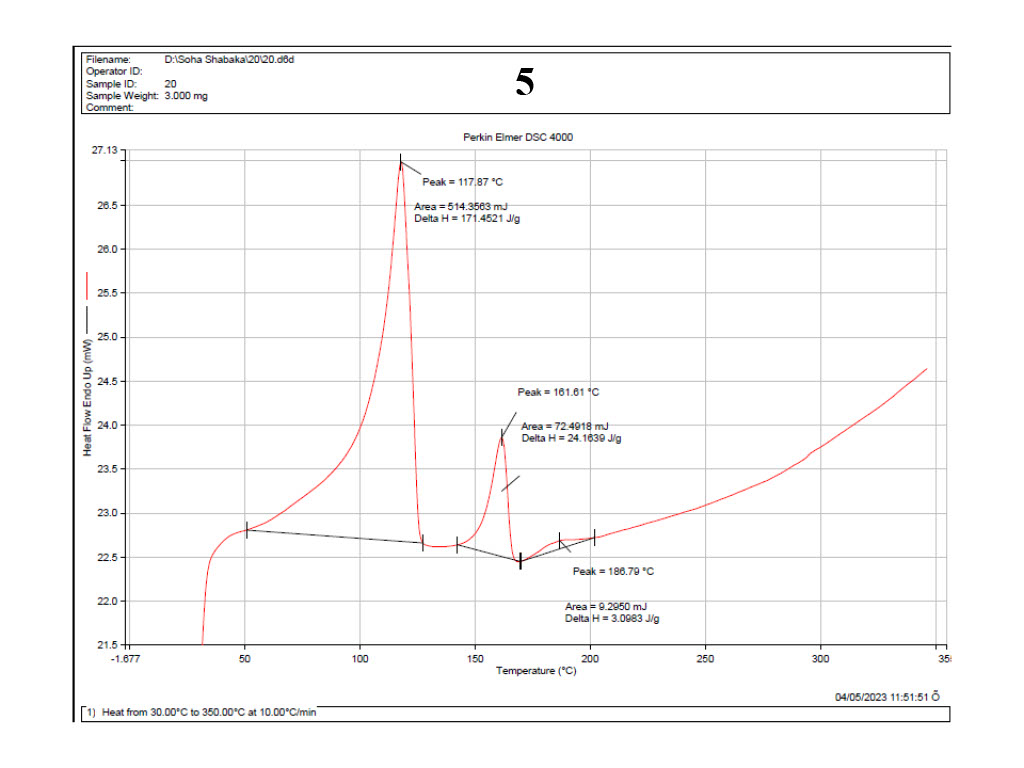

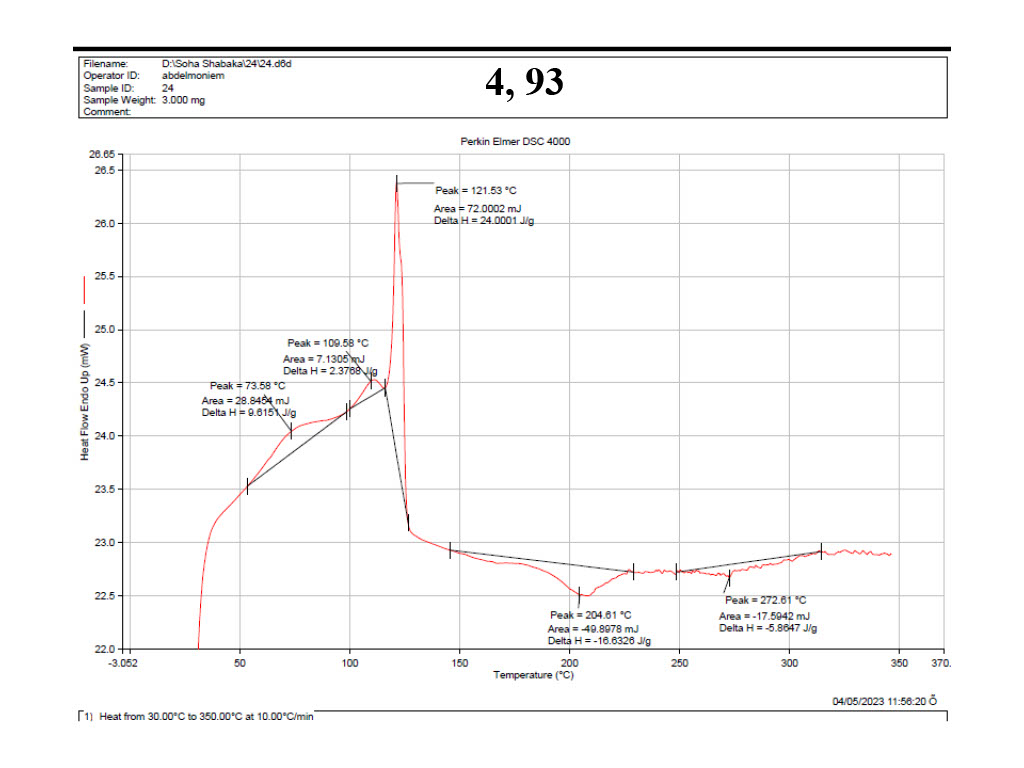

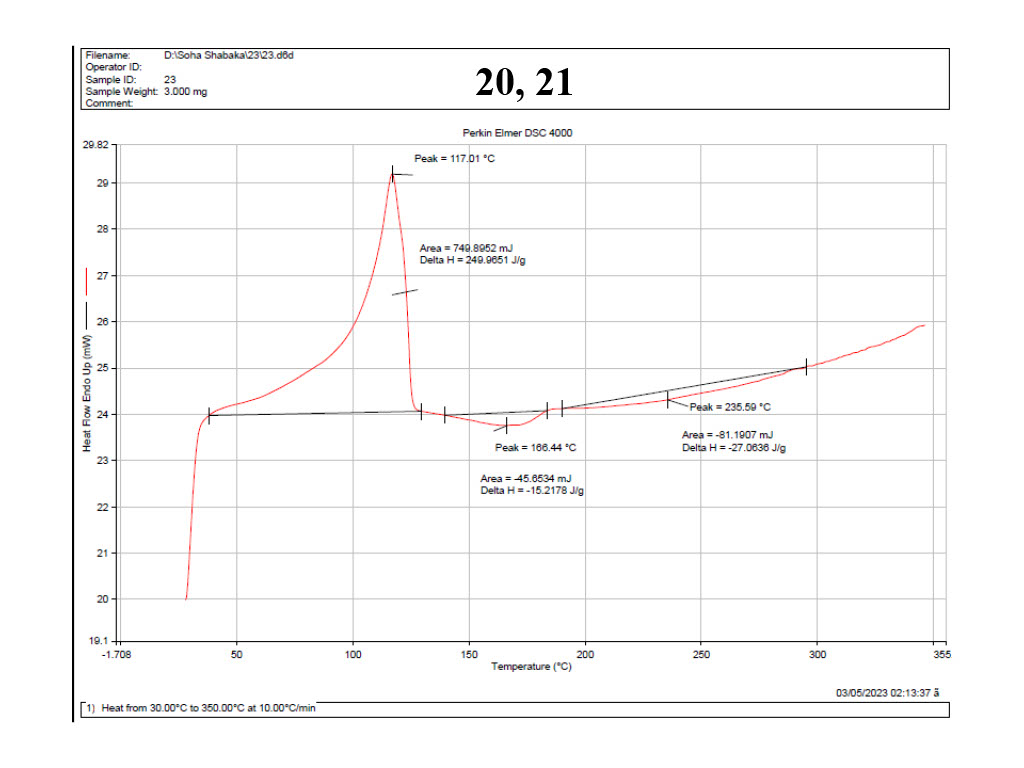

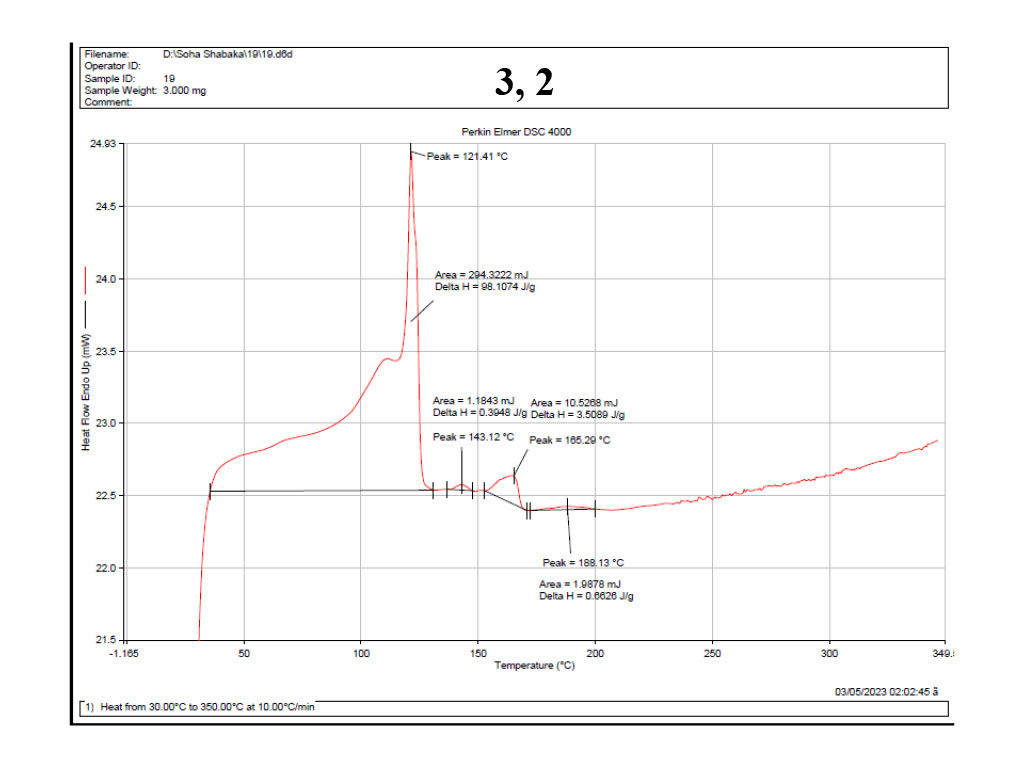

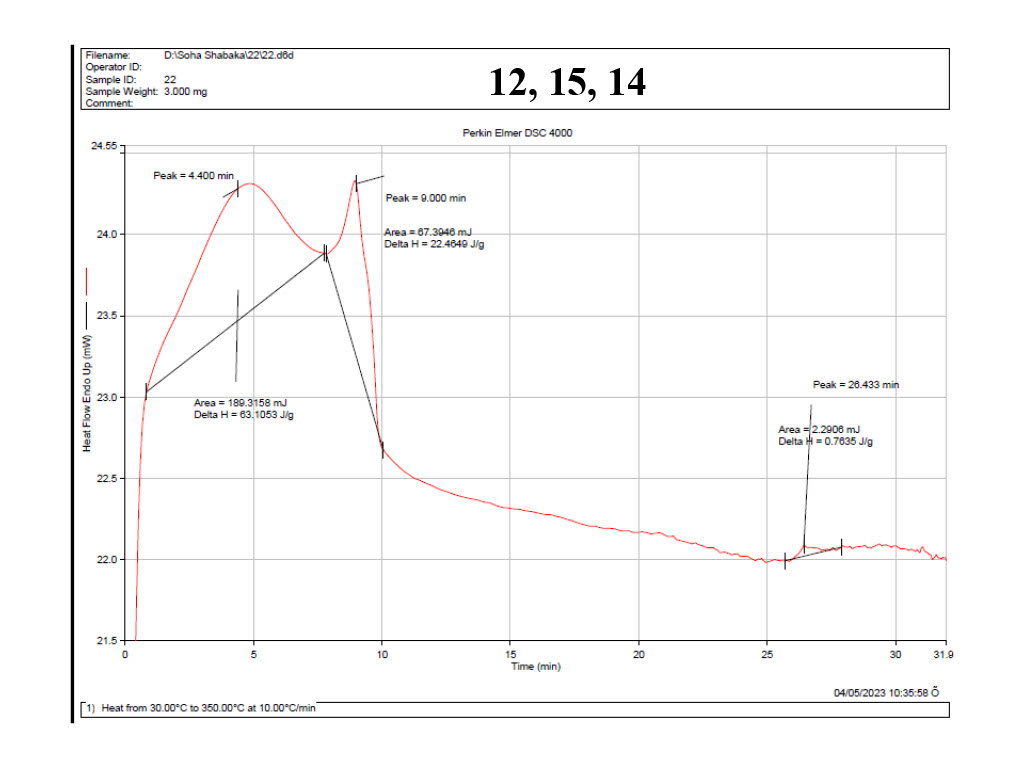

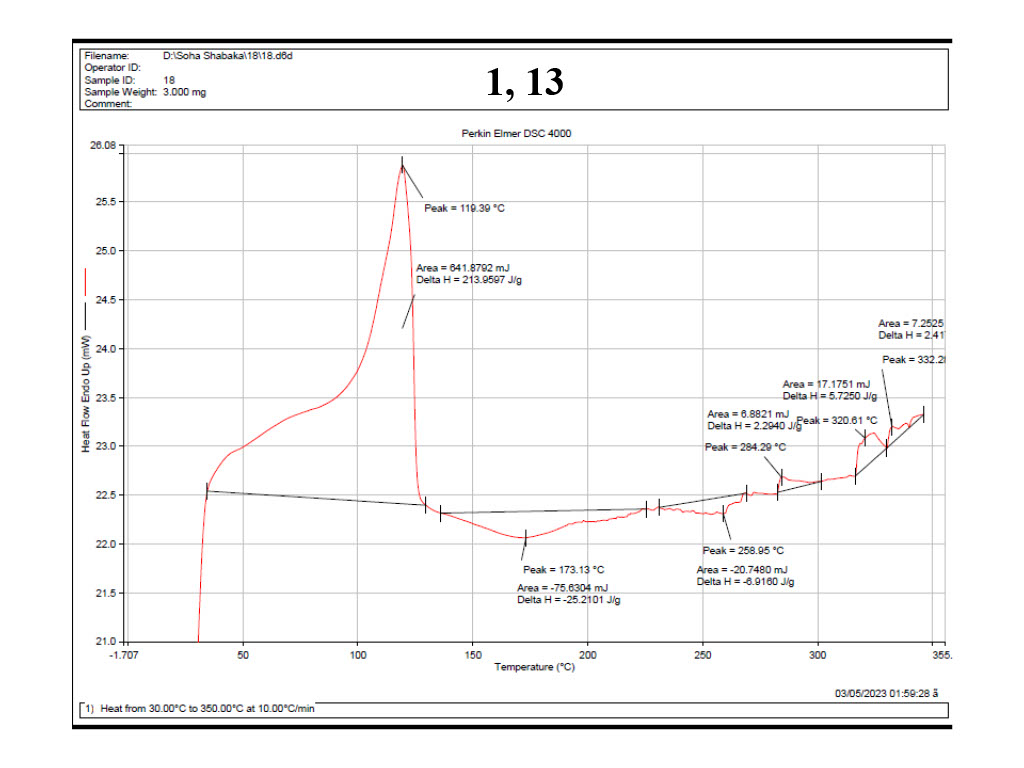


**Table S1.** Weather condition during the study period

| Day | Evapotranspiration  (mm/day) | Temperature  (ºC) | Relative humidity  (%) | Wind speed  (Knot) | Wind direction  (degrees) | Precipitation  (mm) |
| --- | --- | --- | --- | --- | --- | --- |
| 26/10/2022 | 2.647 | 27 | 76 | 5 | 330 | 0 |
| 27/10/2022 | 2.745 | 25 | 71 | 3 | 360 | 0 |
| 28/10/2022 | 2.835 | 25 | 64 | 4 | 340 | 0 |
| 29/10/2022 | 2.678 | 24 | 66 | 4 | 340 | 0 |
| 30/10/2022 | 2.432 | 24 | 66 | 3 | 280 | 0 |
| 31/10/2022 | 2.671 | 24 | 64 | 4 | 280 | 0 |
| 01/11/2022 | 2.382 | 24 | 64 | 5 | 270 | 0 |
| 02/11/2022 | 2.637 | 22 | 58 | 5 | 310 | 0 |
| 03/11/2022 | 2.571 | 23 | 65 | 4 | 300 | 0 |
| 04/11/2022 | 2.507 | 21 | 61 | 3 | 340 | 0 |
| 05/11/2022 | 2.562 | 22 | 56 | 3 | 360 | 0 |
| 06/11/2022 | 2.559 | 22 | 58 | 4 | 260 | 0 |
| 07/11/2022 | 2.469 | 23 | 58 | 7 | 240 | 0 |
| 08/11/2022 | 2.296 | 22 | 58 | 5 | 320 | 0 |
| 09/11/2022 | 2.401 | 23 | 56 | 5 | 310 | 0 |
| 10/11/2022 | 2.051 | 22 | 62 | 4 | 310 | 0 |
| 11/11/2022 | 2.265 | 23 | 58 | 4 | 30 | 9.3 |
| 12/11/2022 | 2.186 | 22 | 75 | 2 | 360 | 11.1 |
| 13/11/2022 | 2.269 | 24 | 71 | 3 | 10 | 0 |
| 14/11/2022 | 1.931 | 22 | 55 | 4 | 340 | 0 |
| 15/11/2022 | 2.043 | 21 | 57 | 4 | 310 | 0 |
| 16/11/2022 | 2.068 | 21 | 65 | 6 | 240 | 0 |
| 17/11/2022 | 2.163 | 22 | 70 | 3 | 270 | 0 |
| 18/11/2022 | NA | 22 | 58 | 3 | 340 | 0 |
| 19/11/2022 | NA | 23 | 66 | 0 | 0 | 0 |
| 20/11/2022 | NA | 23 | 65 | 4 | 70 | 0 |

Table S2 Euclidean distance values, ranking of effects, and weights of factors selected for the water susceptibility model.

| Factor | Euclidean Distance Values | Weight from AHP (%) | Rate of effect |
| --- | --- | --- | --- |
| Waterways | 0 – 0.0358 | 58.8 | 5 |
|  | 0.0358 – 0.0716 |  | 4 |
|  | 0.0717 – 0.107 |  | 3 |
|  | 0.108 – 0.143 |  | 2 |
|  | 0.144 – 0.179 |  | 1 |
| Roads | 0 – 0.0312 | 6.7 | 5 |
|  | 0.0313 – 0.0624 |  | 4 |
|  | 0.0625 – 0.0936 |  | 3 |
|  | 0.0937 – 0.125 |  | 2 |
|  | 0.126 – 0.156 |  | 1 |
| Places (urban areas) | 0 – 0.0679 | 8.1 | 5 |
|  | 0.068 – 0.136 |  | 4 |
|  | 0.137 – 0.204 |  | 3 |
|  | 0.205 – 0.272 |  | 2 |
|  | 0.273 – 0.34 |  | 1 |
| Land uses | 0 – 0.0338 | 10.3 | 5 |
|  | 0.0339 – 0.0676 |  | 4 |
|  | 0.0677 – 0.101 |  | 3 |
|  | 0.102 – 0.135 |  | 2 |
|  | 0.136 – 0.169 |  | 1 |
| Salinity | Low to high | 8.1 | Low effect to high |
| Total suspended solids | Low to high | 8.1 | Low effect to high |
| *1 (very low), 2 (low), 3 (moderate), 4 (high) and 5 (very high)* | | | |

**Table S3.** AHP weight and degree of importance


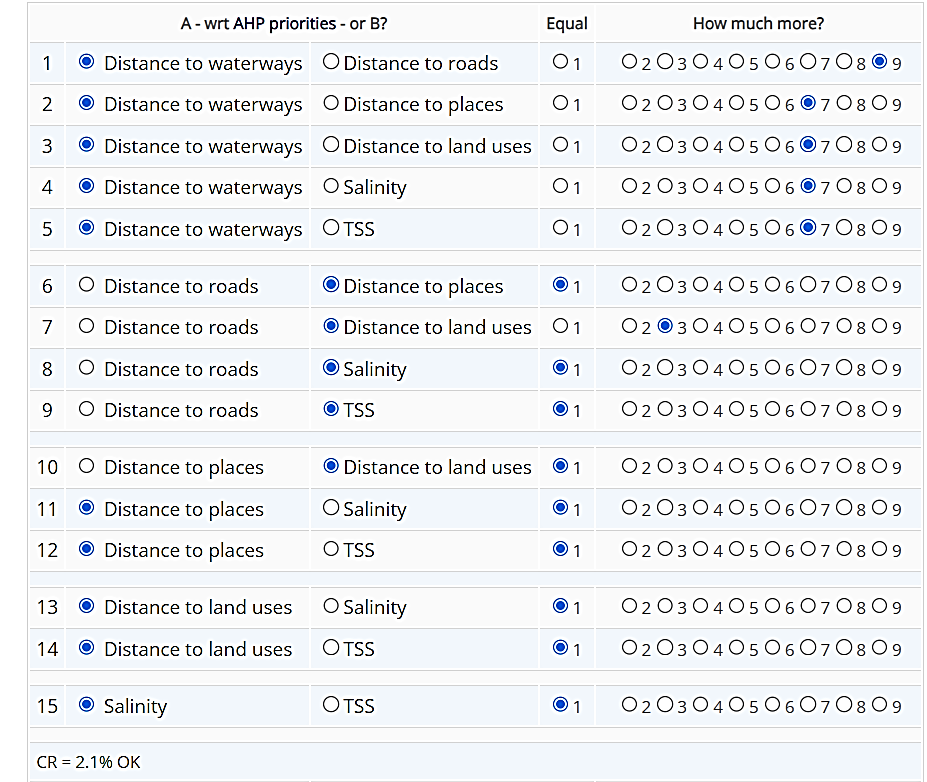


**Table S4.** The resulting weights based on pairwise and principal eigenvector of decision matrix


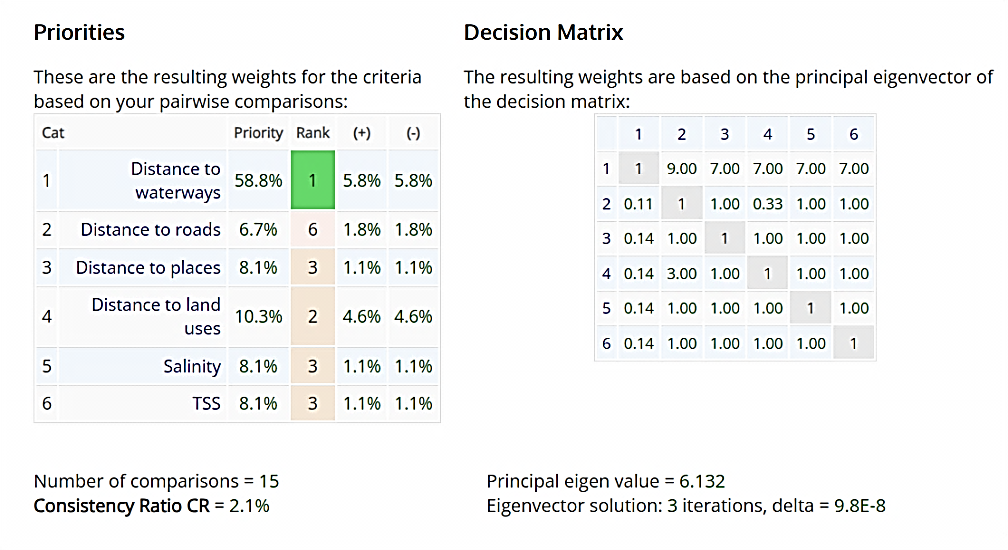


Table S5 Characteristics of MPs in waters from coastal lagoons worldwide . CE: Cellophane. HDPE: high-density polyethylene. LDPE: Low-density polyethylene. NL: Nylon. PA: polyamide. PAA: polyacrylic. PAM: polyacrylamide. PAN: polyacrylonitrile. PE: polyethylene. PET: Polyethylene terephthalate. PES: Polyester. PFTE: polytetrafluoroethylene. PP: polypropylene. PS: Polystyrene. PVC: polyvinyl chloride. PVA: polyvinyl alcohol. PECH: polyepichlorohydrin. PB: polybutadiene. RY: Rayon. IR: polyisoprene. PUR: polyurethane. NBR: acrylonitrile butadiene. EPS: expanded polystyrene. PEP: poly(ethylene-propylene). PEVA: polyethylene vinyl acetate. PVE: polyvinyl ester

| Lagoon loction | Country | Sampling technique | MPs concentration Items m^–3^ | Shape | Color | Polymer composition | Reference |
| --- | --- | --- | --- | --- | --- | --- | --- |
| Acaraí | **Brazil** | Plankton net tow (300µm mesh) | 0.025 | Fibers and fragments | - | - | Lorenzi et al. (2020) |
|  |  |  | 0.0014 - 0.108 | Fragments and filaments | - | PE, PES, PP, PB and PS | Lorenzi et al. (2021) |
| Santo Antônio dos Anjos and Imaruí |  | Plankton net tow (300µm mesh) | 0.40 ±1.03 | Fragments, filaments, and films (soft) | Blue, transparent, red, white, black, yellow, green, and orange | PES, PP, PE and PB | Monteiro et al. (2022) |
| Gangmen | **China** | Seawater sampler (20L) + filter in sieve (48-μm mesh) | 892 | Foams, fibers, films, and fragment | White, brown, blue, transparent, and green | PS | Lin et al. (2021) |
| North Reef |  | water sampler + filtration (0.45 µm) | 9400 | Fibers, fragments, granules, and films | Red, black, blue, green, grey, transparent, brown, and yellow | RY, PET, NL, PAM, PE, PECH, PVA, PVC | Ding et al. (2019) |
|  |  | water sampler + filtration (0.45 µm) | 45200 |  |  |  |  |
| Yongle Atoll |  |  | 19200 |  |  |  |  |
| Passu Keah |  |  | 4600 |  |  |  |  |
| Xincun |  | water sampler (1L) | 81600 - 34300 | Fibers, fragments, and films | Transparent, blue, black, red, green, yellow, and purple | CE, PP, PE, and PS | Wei et al. (2022) |
|  |  | water sampler (20L) + filter in sieve (48-μm mesh) | 154 | Foams, fibers, films, and fragment | White, brown, blue, transparent, and green | PS | Lin et al. (2021) |
| Ciénaga Grande de Santa Marta | **Colombia** | Metal bucket (100L) + filter in sieve (300 µm mesh) | 44- 51 | Fibers, fragments, films, foams, and granules | Colorless, white, blue, black, green, yellow, brown, and red | PE, PP, HDPE, PS | Garcés-Ordóñez et al. (2022) |
| Sakumo II | **Ghana** | Glass bottles | 90000 | - | Brown, black, translucent, and white | - | Gbogbo et al. (2020) |
| Rio Lagartos | **Mexico** | Zooplankton net tow (200 µm mesh) | 0.6 | Fibers, fragments, and films | - | - | Quesadas-Rojas et al. (2021) |
| Lagos | **Nigeria** | Direct collect (700 mL) | 208000 | Fibers, fragments, and films | Black, blue, transparent, grey, green, purple, red, turquoise, white, and yellow | P, PE, and PES | Olarinmoyec et al. (2020) |
| Ahe | **French Polinesia** | Planktonic net (40 µm-mesh) | 83 | Fibers and fragments | Black/grey, orange, yellow, red, brown, and blue | PE, IR, and PS | Gardon et al. (2021) |
| Manihi |  |  | 84 |  |  | PE, IR, and PVC |  |
| Takaroa |  |  | 135 |  |  | PE, PES, and IR |  |
| Ahe |  | Standard manta trawl (300 µm-mesh) | 3 |  | Black/grey, blue, red, yellow, brown, and orange | PE, PU, and PS |  |
| Manihi |  |  | 3 |  |  | PE, PP, and PES |  |
| Takaroa |  |  | 1 |  |  | PES, PE, and PA |  |
| Aveiro | **Portugal** | Glass bottles | 18000 | - | - | - | Prata et al. (2020) |
| Bundala | **Sri Lanka** | Plankton net tow (80µm mesh) | 0 | - | - | - | Sevwandi et al. (2021) |
| Embilikala |  |  | 0 |  |  |  |  |
| Malala |  |  | 0 |  |  |  |  |
| Bizerte | **Tunisia** | Water pump (20L) + stainless-steel filter (300µm mesh) | 400 | Fibers, films, and fragments | Clear, blue, white, red, green, yellow, and black | PE, PP and CE | Wakkaf et al. (2020b) |
|  | **Tunisia** |  | 453 | Fibers, fragments, and films | White, transparent, blue, black, green, red, and yellow | PE, PP, PET, CE, NL, and PS | Wakkaf et al. (2020a) |
| Küçükçekmece | **Turkey** | Teflon pump (10 L) + Stainless steel sieve (50µm mesh) | 33000 | Fragments, fibers, lines, and films | Blue, red and green | PE | Faruk et al. (2021) |
| Lagoons north of Florida Bay | **USA** | Integrating sample pole (PVC) | 24000000 | Fragments | Blue | PS | Badylak et al. (2021) |
| Barnes Sound |  |  | 20000000 |  |  |  |  |
| Mosquito |  | Plastic bottles (1-L) | 15600 - 33900 | Fibers, fragments, and beads | - | - | Waite et al. (2018) |
| Thi-Nai | **Vietnam** | Plankton net tow (80µm mesh) | 3.2-4.1 | Fibers and fragments | - | PE, PP, PES, and PS | Strady et al. (2021) |

**References mentioned in Table S5**

- Badylak et al. (2021) DOI: 10.1038/s41598-021-85388-y
- Ding et al. (2019) DOI:10.1021/acs.est.9b01452
- Faruk et al. (2021) DOI:10.1016/j.envpol.2020.115801
- Garcés-Ordóñez et al. (2022) DOI:10.1016/j.scitotenv.2022.154643
- Gardon et al. (2021) DOI:10.1016/j.jhazmat.2021.126396
- Gbogbo et al. (2020). DOI:10.1007/s10661-020-8175-8
- Lin et al. (2021). DOI:10.1016/j.gr.2021.11.008
- Lorenzi et al. (2020). DOI:10.1007/s11356-020-07708-5
- Lorenzi et al. (2021). DOI:10.1016/j.marpolbul.2021.112644
- Monteiro et al. (2022) DOI:10.1016/j.marpolbul.2022.113648
- Olarinmoyec et al. (2020). DOI:10.3390/geosciences10120494
- Prata et al. (2020). DOI:10.3390/w12041219
- Quesadas-Rojas et al. (2021).DOI:10.1016/j.scitotenv.2021.145803
- Sevwandi et al. (2021). DOI:10.1016/j.marpolbul.2021.112462
- Strady et al. (2021). DOI:10.1016/j.marpolbul.2020.111870
- Waite et al. (2018). DOI:10.1016/j.marpolbul.2018.02.026
- Wakkaf et al. (2020a). DOI:10.1016/j.marpolbul.2020.111625
- Wakkaf et al. (2020b). DOI:10.1016/j.marpolbul.2020.111355
- Wei et al. (2022). DOI:10.3389/fenvs.2022.829942
